# Targeting Immune Checkpoint Inhibitors for Non-Small-Cell Lung Cancer: Beyond PD-1/PD-L1 Monoclonal Antibodies

**DOI:** 10.3390/cancers17050906

**Published:** 2025-03-06

**Authors:** Nicolas Roussot, Courèche Kaderbhai, François Ghiringhelli

**Affiliations:** 1 Department of Medical Oncology, Centre Georges-François Leclerc, 21000 Dijon, France; cgkaderbhai@cgfl.fr (C.K.); fghiringhelli@cgfl.fr (F.G.); 2 Cancer Biology Transfer Platform, Centre Georges-François Leclerc, 21000 Dijon, France; 3 Centre de Recherche INSERM LNC-UMR1231, Team TIRECs: Therapies and Immune REsponse in CancerS, 21000 Dijon, France; 4 UFR Sciences de Santé, Université de Bourgogne, 21000 Dijon, France

**Keywords:** non-small-cell lung cancer, NSCLC, immune checkpoint, immunotherapy, PD-1, PD-L1, clinical trial

## Abstract

Non-small-cell lung cancer (NSCLC) remains a leading cause of cancer-related mortality worldwide. Immunotherapy targeting the PD-1/PD-L1 axis has revolutionized treatment, providing durable responses in a subset of patients. However, with fewer than 50% of patients achieving significant benefits, there is a critical need to expand therapeutic strategies. This review explores emerging targets in immune checkpoint inhibition beyond PD-1/PD-L1, including CTLA-4, TIGIT, LAG-3, TIM-3, NKG2A, and CD39/CD73. We highlight the biological basis of CD8 T cell exhaustion in shaping the antitumor immune response. Novel therapeutic approaches targeting additional inhibitory receptors (IR) are discussed, with a focus on their distinct mechanisms of action and combinatory potential with existing therapies. Despite significant advancements, challenges remain in overcoming resistance mechanisms and optimizing patient selection. This review underscores the importance of dual checkpoint blockade and innovative bispecific antibody engineering to maximize therapeutic outcomes for NSCLC patients.

## Simple Summary

This review provides the latest data regarding the clinical development of new immune checkpoints inhibitors for the treatment of non-small-cell lung cancer, beyond the PD-1 and PD-L1 targets.

## 1. Introduction

Non-small-cell lung cancer (NSCLC) remains a leading cause of cancer-related mortality worldwide [1]. Despite advancements in conventional therapies, metastatic NSCLC (mNSCLC) continues to pose significant challenges [2]. In recent years, immunotherapy has emerged as a transformative approach, revolutionizing the cancer treatment landscape. By harnessing the body’s immune system to combat cancer cells, immunotherapy offers a promising avenue for improving patient outcomes [3]. In patients without oncogene addiction [4], the first-line regimen for metastatic disease was transformed by the use of anti-PD-1/PD-L1 (aPD-1/aPD-L1) antibodies (mAbs) [5,6,7,8,9]. These agents target the programmed cell death protein 1 (PD-1) and its ligand (PD-L1), which are expressed on cancer and immune cells. By blocking the PD-1/PD-L1 pathway, these antibodies help to restore the immune system’s ability to recognize and attack tumor cells [10,11,12,13]. This concept is based on the capacity of cancer to trigger a particular differentiation of CD8 T cells, called the exhaustion program [14,15]. Antitumor T cells, after recognition of their targets, acquire the exhaustion program, marked by the expression of the transcriptional factor TOX1 and the expression of the checkpoint inhibitor PD-1, as well as the expression of multiple additional inhibitory receptors (IRs) [16,17,18,19].

The development of aPD-1/PD-L1 therapies has marked a significant milestone in the treatment of mNSCLC. Based on PD-L1 expression on cancer cells (tumor positive score (TPS)) patients are treated with aPD-1/PD-L1 therapies alone or in combination with chemotherapies [4]. These treatments have demonstrated remarkable efficacy in a subset of patients, leading to durable response and improved survival. However, it is important to note that fewer than 50% of patients benefit from immunotherapy [2]. Factors such as PD-L1 expression [20], tumor mutational burden (TMB) [21,22,23], and immune cell composition [24,25,26,27] can influence treatment response, suggesting that better patient selection could be achieved. In addition, to address the limitations of current immunotherapy approaches, ongoing research is focused on developing complementary therapies. These may include combinations with chemotherapy, targeted therapies, or other immunotherapeutic agents. In this review, we report current clinical research on combinatory treatments that involved dual checkpoint inhibitor blockade.

## 2. CD8 T Cell Exhaustion

In the context of cancer or chronic infection, and contrary to acute infection or vaccination, CD8 T cells within the tumor are permanently exposed to cancer cells or to pathogens, thus preventing complete antigen clearance. With such antigen persistence, the T cell receptor (TCR) undergoes chronic signaling that mediates a path of differentiation, gradually leading to CD8 T cell exhaustion (reviewed in references [14,15,28,29]). Although heterogeneous, exhausted CD8 T (CD8 Tex) cells are characterized by a progressive loss of effector functions and proliferative potential, impaired responsiveness to homeostatic cytokines, a shift in the metabolic program, altered epigenetic and transcriptional profile, and high expression of IRs. As a consequence of permanent TCR stimulation, activated CD8 T cells longitudinally progress from precursor CD8 Tex (TCF1, EOMES, and TOX expression) to progenitor CD8 Tex (self-renewal ability) to intermediate CD8 Tex (loss of TCF1, sustained TOX expression) and finally to terminal CD8 Tex cells (sustained TOX expression, high IR expression, low cytokine production).

Biologically, the balance between the two transcription factors TCF-1 [30] (encoded by *Tcf7*) and TOX [31] (encoded by *Tox*) mediates entrance into and progression of the exhaustion program. In the early phase of exhaustion, progenitor CD8 Tex cells that express TCF-1 conserve proliferative potential. It is now clear that the cellular proliferation increase and the CD8 Tex cell reinvigoration observed after PD-1 blockade result from the effects on this progenitor CD8 Tex subset [32,33,34]. As the exhaustion program progresses, progenitor CD8 Tex cells lose TCF-1 and acquire TOX to become intermediate CD8 Tex, then terminal CD8 Tex cells. TOX might be considered as the tipping point of exhaustion, since its expression is sufficient to promote all key features of CD8 Tex cells [16,18,19], notably the transcriptional regulation and chromatin remodeling that orchestrates the epigenetic landscape of CD8 Tex cells [17]. This epigenetic imprint further limits the anti PD-1 ability to restore effector or memory T cell phenotypes [35,36,37]. Indeed, transcriptomic and epigenetic characterization of CD8 T cells at a single-cell scale clearly distinguished distinct epigenetic features for the canonical CD8 genes *Tcf7*, *Tox*, and also *Pdcd1* (encoding the IR PD-1) between CD8 T effector, CD8 T memory, and CD8 Tex subsets. To go deeper into the CD8 Tex granularity, chromatin accessibility analyses revealed the existence of distinct epigenetic subsets among the CD8 Tex population. Regarding the impact of immunotherapy within these distinct CD8 Tex subsets, PD-L1 blockade accelerates the differentiation from progenitor CD8 Tex to terminal CD8 Tex cells [38]. Recent studies have deciphered the role of distinct CD8 Tex IRs, demonstrating that IR signaling is not redundant and may synergize to regulate exhaustion features [39,40,41]. Indeed, while PD-1 restrains cellular proliferation, LAG-3 reduces cytokine production, sustains TOX expression, and upregulates the inhibitory CD94/NKG2A receptor through a LAG-3/TOX/CD94/NKG2 axis [42]. Such biological discrepancies between IRs may explain the synergic effect observed with PD-1 and LAG-3 co-blockade [43], notably in NSCLC [44,45]. At the proteome scale, PD-1-positive LAG-3-positive CD8 Tex display a specific enrichment in ubiquitination pathways, especially the E3 ubiquitin ligase Casitas B cell lymphoma (Cbl-b) [46,47].

Pan-cancer analysis of T cells across 16 human cancer types uncovered a T cell transcriptional state characterized by a stress response signature. These stressed CD8 T cells are detectable across distinct cancer types and are enriched in tumors non-responsive to anti-PD1 therapy, notably in NSCLC [48]. Recently, ADRB1, the b1 adrenergic receptor, has been described as a new immune checkpoint and exhaustion marker, as it is expressed by terminal CD8 Tex, but not progenitor CD8 Tex cells, and it promotes exhaustion through an ADRB1-cAMP-CREM pathway, thus suggesting that stress-associated catecholamines participate in CD8 T cell exhaustion [49,50]. Apart from the sympathetic-induced stress response, mechanical stress due to a dense stroma in the tumor microenvironment (TME) induces the transcription factor Osr2 and also mediates CD8 T cell exhaustion [51]. These emerging data uncover the existence of unexpected signaling pathways in the exhaustion program. In the past decade, the main strategy developed to reinvigorate CD8 Tex cells has been to engineer mAb blocking IRs. This review further focuses on the targeting of IRs, namely CTLA-4, LAG-3, TIGIT, TIM-3, CD94/NKG2A, and CD39/CD73.

## 3. CTLA-4 Targeting

**Figure 1:**
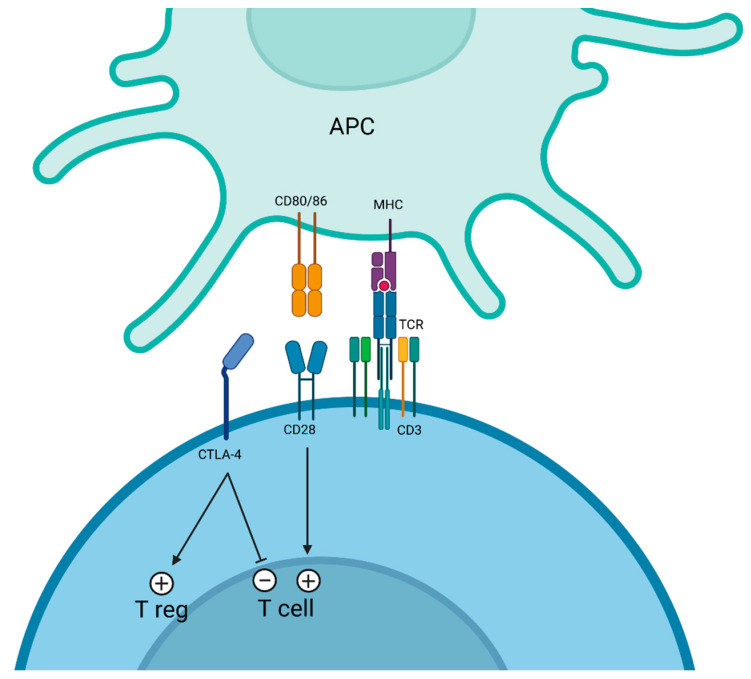
CTLA-4 mechanism of action. APC: antigen-presenting cell.

Cytotoxic T lymphocyte-associated protein 4 (CTLA-4) is a pivotal immune checkpoint [52] that plays a central role in regulating T cell activation and maintaining immune homeostasis [53]. It is expressed predominantly on activated T cells and regulatory T cells (Tregs) [54]. CTLA-4 competes with the co-stimulatory receptor CD28 for binding to B7 ligands (CD80 and CD86) on antigen-presenting cells (APC), thereby transmitting an inhibitory signal that limits T cell proliferation and cytokine production [55] (Figure 1). By dampening T cell responses, CTLA-4 prevents autoimmunity but also facilitates tumor immune evasion. Inhibiting CTLA-4 has been a groundbreaking strategy in cancer immunotherapy, aiming to unleash T cell-mediated antitumor immunity [54]. The first-in-class anti-CTLA-4 (aCTLA-4) [56] mAb, ipilimumab, demonstrated durable clinical benefits in melanoma, marking a paradigm shift in oncology and earning FDA approval in 2011. Subsequently, other CTLA-4 targeting agents, such as tremelimumab, have been developed and explored for use in diverse malignancies [57].

The mechanisms of action seem to be multifunctional and involve the capacity of CTLA-4 blockade to improve effector T cell function and increase the proliferation and activation of effector CD4+ and CD8+ T cells. This leads to increased cytokine production and cytotoxic activity, promoting tumor cell eradication. CTLA-4 is preferentially expressed on immunosuppressive Tregs [58]. Anti-CTLA-4 therapies may contribute to antitumor efficacy through the selective depletion of intratumoral Tregs in an Fc receptor-dependent manner [59]. Nevertheless, aCTLA-4 mAbs that lack a crystallizable fragment (Fc) still induce tumor regression, thus suggesting Fc-independent functions [60,61]. CTLA-4 inhibition expands the repertoire of T cells capable of recognizing diverse tumor antigens [62]. This increase in clonal diversity is critical for robust and sustained antitumor responses. Finally, by blocking CTLA-4, these therapies restore the CD28-mediated co-stimulatory signals required for optimal T cell activation and survival. Published results from clinical trials with aCTLA-4 therapies are summarized in Table 1.

### 3.1. Ipilimumab

Ipilimumab is a fully human IgG1 monoclonal antibody and was the first CTLA-4 inhibitor to demonstrate significant survival benefits in advanced melanoma. Early clinical trials reported that ipilimumab improved overall survival (OS) by several months compared to standard treatments, with a subset of patients achieving long-term remission. Ipilimumab induces durable responses, with some patients achieving survival beyond 10 years [56]. In addition to melanoma, ipilimumab has shown efficacy in other cancers, including NSCLC [63], renal cell carcinoma [64], mesothelioma [65], hepatocellular carcinoma [66], and microsatellite instable (MSI-high) colorectal cancer [67], often in combination with other checkpoint inhibitors like aPD-1 agents [68]. While durable responses have been observed with immune checkpoint inhibitor (ICI) monotherapies, such as ipilimumab, only about 20% of patients respond to single-agent ipilimumab in melanoma. This limitation has driven research into combination therapies to enhance efficacy. Clinically, combining checkpoint inhibitors—such as aCTLA-4 and aPD-1/PD-L1—has shown higher response rates compared to monotherapy with either agent across multiple tumor types. Notably, this combination has demonstrated improved OS in melanoma in comparison to aPD-1 alone or ipilimumab alone [69,70]. Additionally, aCTLA-4 and aPD-1 combination therapy has also exhibited antitumor activity in patients who failed aPD-(L)1 monotherapy, suggesting its potential to overcome adaptive resistance mechanisms in select cases. However, since not all patients respond to the combination therapy, identifying novel mechanisms underlying primary and adaptive resistance is essential to guide rational therapeutic strategies. In a first-line setting for mNSCLC, the CheckMate-9LA [71] trial tested nivolumab plus ipilimumab with two cycles of chemotherapy vs. chemotherapy alone. Immunotherapy strongly improved OS (hazard ratio (HR), 0.73; 95% confidence interval (CI), 0.62–0.85; 5-year OS rates, 18% vs. 11%), regardless of tumor PD-L1 expression or histology or presence of baseline brain metastases. The five-year duration of response (DOR) rate was 19% [72]. The CheckMate-227 [63] trial, which tested nivolumab plus ipilimumab without chemotherapy vs. chemotherapy for PD-L1-positive patients, also demonstrated that the combination of aCTLA-4 with aPD-1 improved OS in this population [73]. These data support the long-term efficacy of this combination for mNSCLC.

### 3.2. Tremelimumab

Tremelimumab is a human IgG2 anti-CTLA-4 mAb, as developed to address the toxicity concerns associated with ipilimumab. Though initial studies in melanoma did not meet primary endpoints [74], tremelimumab has shown promise in combination regimens. Currently, tremelimumab, combined with the aPD-L1 mAb durvalumab, is approved for unresectable hepatocellular carcinoma in a first-line regimen for metastatic disease [75]. In contrast, in NSCLC, the POSEIDON trial failed to demonstrate a significant improvement in OS with the combination of chemotherapy plus durvalumab and tremelimumab [76]. The particularity of the CTLA-4 blockade is the dose effect of the drug. Indeed, aCTLA-4 therapies are associated with significant toxicity, including colitis, dermatitis, and endocrine disorders. These immune-related adverse events (irAEs) result from immune overactivation and are usually dose-dependent [77]. Strategies such as dose reduction (e.g., 1 mg/kg vs. 3 mg/kg for ipilimumab) and intermittent dosing have been explored to mitigate these effects [78]. However, the efficacy of the CTLA-4 blockade used in monotherapy or in combination with aPD-1/aPD-L1 also seems dose-dependent.

### 3.3. Botensilimab

Botensilimab is a promising Fc-enhanced aCTLA-4 mAb designed to address the limitations of the conventional CTLA-4 blockade, with the aim of improving treatment efficacy in “cold” or refractory tumors. Fc-optimized mAbs are being engineered to enhance Treg depletion, while minimizing systemic toxicity. Fc-optimized antibodies selectively deplete intratumoral Tregs, improving therapeutic efficacy. Botensilimab demonstrates enhanced binding to activating Fcγ receptors (FcγRs) on APC and natural killer (NK) cells, optimizing T cell priming, activation, and memory formation. It also promotes intratumoral regulatory T cell depletion through mechanisms like antibody-dependent cellular cytotoxicity (ADCC) and phagocytosis [79,80]. This treatment showed promising efficacy in phase I studies in immune refractory tumor and in microsatellite stable colorectal cancer known to be resistant to classical immunotherapies [81]. Trials are in progress in other solid tumors with only a few results for NSCLC [80].

Another strategy involves the use of bispecific antibodies (bsAbs) targeting both aCTLA-4 and aPD-(L)1.

### 3.4. Cadonilimab (AK-104)

Cadonilimab is a humanized bsAb that binds concurrently to PD-1 and CTLA-4 with high affinity on tumor-infiltrating lymphocytes (TILs), thereby enhancing antitumor efficacy and also safety [82]. For mNSCLC, the AK104-202 study [83], a multicenter, open-label clinical trial, enrolled 53 patients and evaluated the safety and efficacy of cadonilimab. The patients were split into three groups. Group A consisted of patients who had failed platinum-based doublet chemotherapy and were immunotherapy naïve; Group B and Group C comprised patients who had failed platinum-based doublet chemotherapy and showed primary or acquired resistance to ICI, respectively. The primary endpoint was not met; mOS was 19.61, 4.93, and 13.16 months in Groups A, B, and C, respectively. Only 10% of the patients in Group A had a response, while the ORR was 0% in Groups B and C. Clinical trials with cadonilimab are currently ongoing, such as the AK104-207 (NCT04647344), the AK104-208 (NCT04646330), and the LungCadX studies [84]. Although this drug seems to be less efficient for patients that have already experienced resistance to immunotherapy, the results of these trials are expected to bring new insights regarding cadonilimab’s usefulness in NSCLC treatment strategies.

### 3.5. Erfonrilimab (KN-046)

Erfonrilimab is an IgG-like bsAb that specifically binds to PD-L1 and CTLA-4. Erfonrilimab showed significant antitumor efficacy in a melanoma xenograft mouse model and in a colon cancer syngeneic mouse model. After the phase I study with erfonrilimab, involving enrolled patients with advanced solid tumors, demonstrated safety and a promising antitumor efficacy [85], a phase II study was conducted in patients with mNSCLC in the first-line setting. The blinded independent review committee (BIRC)-assessed ORR was 46%, with a median progression-free survival (PFS) of 5.8 months and a median OS of 26.6 months [86].

### 3.6. Volrustomig (MEDI5752)

Volrustomig is also an aPD-1/aCTLA-4 bsAb with data reported from a phase Ib/II trial in the first-line setting for mNSCLC. At the 1500 mg dose, with chemotherapy, this drug induced an ORR of 50.0% vs. 47.6% in patients assigned to pembrolizumab plus chemotherapy. The median PFS (15.1 vs. 8.9 months) and OS (not reached vs. 16.5 months) also favored the volrustomig arm [87]. At the 750 mg dose, with chemotherapy, the ORRs were 43.7% and 65.0% in non-squamous and squamous mNSCLC, respectively [88]. Volrustomig with chemotherapy is currently being evaluated in the first-line setting of PD-L1< 50% mNSCLC in the randomized phase III eVOLVE-Lung02 study, versus pembrolizumab with chemotherapy [89].

Additional bsAbs are under development, such as lorigerlimab and vudalimab.

**Table 1:** Published data from clinical trials with anti-CTLA-4 monoclonal antibodies or bispecific antibodies in non-small-cell lung cancer.

aCTLA-4 mAb	Type	Study	Phase	Setting	N	Intervention	1st Endpoint	Results with aCTLA-4	Ref
**Ipilimumab**	human IgG1κ	CM-9LA	III	Metastatic, 1st line	719	Nivo (aPD-1) +ipi + CT vs. CT	OS in overall population	mOS 15.8 mo with nivo + ipi + CT vs. 11.0 mo with CT (HR 0.66; 95% CI, 0.55–0.80), 5 y OS 18% with nivo + ipi + CT vs. 11% with CT mPFS 6.7 mo with nivo + ip + CT vs. 5.3 mo with CT (HR 0.70; 95% CI, 0.60–0.83) Grade ≥ 3 TRAE 47% with nivo + ipi + CT	[71,72]
		CM-227	III	Metastatic, 1st line	1189	Nivo + ipi vs. nivo vs. CT	OS in PD-L1 ≥ 1% of nivo + ipi vs. CT	mOS 17.1 mo with nivo + ipi vs. 14.9 mo with CT (*p* = 0.007) mPFS 5.1 mo with nivo + ipi vs. 5.6 mo with CT Grade ≥ 3 TRAE 32.8% with nivo + ipi vs. 19.4% with nivo	[63,64,65,66,67,68,69,70,71,72,73]
**Tremelimumab**	human IgG2a	POSEIDON	III	Metastatic, 1st line	1013	Treme + durva (aPD-L1) + CT vs. durva + CT vs. CT	PFS and OS of durva + CT vs. CT	mOS 13.3 mo with treme + durva + CT vs. 11.7 mo with CT (HR, 0.86; 95% CI, 0.72 to 1.02; *p* = 0.758) mPFS 5.5 mo with treme + durva + CT vs. 4.8 mo with CT (HR, 0.74; 95% CI, 0.62 to 0.89; *p* = 0.0009) Grade ≥ 3 TRAE 51.8% with treme + durva + CT vs. 44.6% with durva + CT	[76]
**Botensilimab**	Fc-enhanced human IgG1	Chand et al.	Ia/b	Metastatic, refractory	3 with NSCLC	Boten +/− balsti (aPD-1)	Safety	ORR = 67% Grade ≥ 3 TRAE 33% with boten vs. 26% with boten + balsti	[80]
**aCTLA-4 bsAb**	**Type**	**Study**	**Phase**	**Setting**	**N**	**Intervention**	**1st Endpoint**	**Results with aCTLA-4**	**Ref**
**Cadonilimab (AK-104)**	CTLA-4/PD-1	AK104-202	Ib/II	Metastatic, Co A: 2nd line, ICI-naïve Co B: 2nd line, primary resistance to CT-ICI Co C: 2nd line, secondary resistance to CT-ICI	30 (Co A) 7 (Co B) 16 (Co C)	Cadon	ORR	ORR 10% in Co A, 0% in Co B and C mOS 19.61 m in Co A, 4.93 m in Co B, 13.16 m in Co C Grade ≥ 3 TRAE 11.3%	[83]
**Erfonrilimab (KN-046)**	CTLA-4/PD-1	Zhao et al.	II	Metastatic, 1st line	87	Erfon + CT	ORR, DoR	ORR = 46.0% mDoR = 8.1 mo mPFS 5.8 mo, mOS 26.6 mo Grade ≥ 3 TRAE 66.7%	[86]
**Volrustomig (MEDI5752)**	CTLA-4/PD-1	Ahn et al., Spigel et al.	Ib/II	Metastatic, 1st line	139	Volru 1500 mg + CT vs. volru 750 mg + CT vs. pembro (aPD-1) + CT	ORR	ORR 50.0% with volru 1500 mg + CT vs. 47.6% with pembro + CT mPFS 15.1 mo with volru 1500 mg + CT vs. 8.9 mo with pembro + CT mPFS NR with volru 1500 mg + CT vs. 16.5 mo with pembro + CT ORR with volru 750 mg + C: 43.7% in non-squamous, 65.0% in squamous NSCLC No safety data	[87,88]

mAb: monoclonal antibody; N: number of patients included; nivo: nivolumab; ipi: ipilimumab; CT: chemotherapy; OS: overall survival; mOS: median overall survival; mo: months; HR: hazard ratio; CI: confidence interval; mPFS: median progression-free survival; 5 y: 5-year; TRAE: treatment-related adverse events; treme: tremelimumab; durva: durvalumab; PFS: progression-free survival; boten: botensilimab; balsti: balstilimab; ORR: objective response rate; bsAb: bispecific antibody; Co: cohort; cadon: cadonilimab; ICI: immune checkpoint inhibitor; erfon: erfonrilimab; DoR: duration of response; mDoR: median duration of response; pembro: pembrolizumab; volru: volrustomig.

## 4. TIGIT Targeting

**Figure 2:**
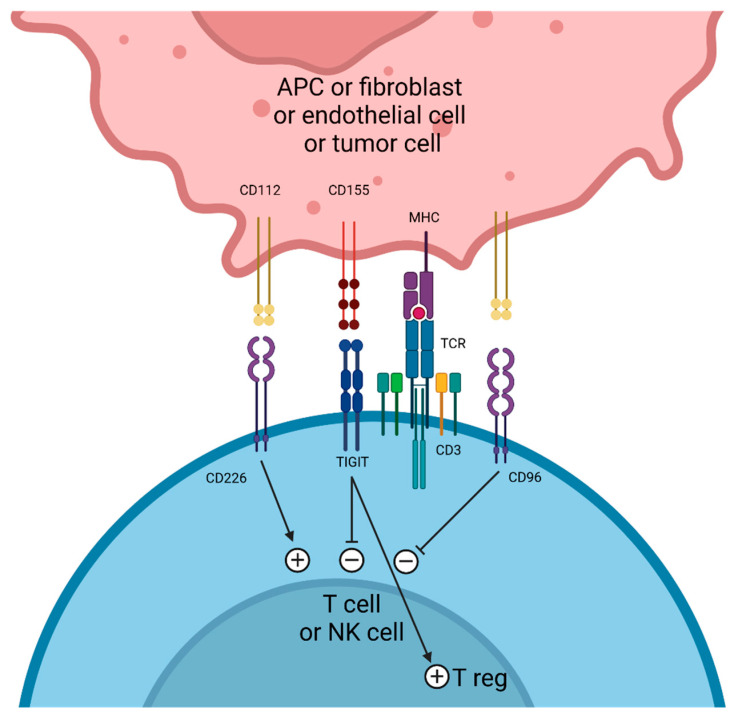
TIGIT mechanism of action. APC: antigen-presenting cell.

TIGIT (also known as WUCAM, VSTM3, or Vsig9) is an inhibitory molecule expressed on CD4 and CD8 T lymphocytes, Treg lymphocytes, and NK lymphocytes [90,91,92]. The expression of TIGIT by Treg lymphocytes is involved in their immunosuppressive functions. Tregs expressing TIGIT present an activated phenotype and express a high level of immunosuppressive markers, such as FoxP3, CTLA-4, PD-1, TIM-3, and LAG-3 [93]. TIGIT binds primarily to the CD155 receptor, also known as the polio virus receptor (PVR), which is expressed on monocytes, dendritic cells (DCs), fibroblasts, endothelial cells, and tumor cells from many cancers [94]. TIGIT also binds, albeit with less affinity, to CD112, also known as Nectin-2, which is expressed on DCs, monocytes, and numerous tumor cell subtypes. TIGIT ligands (CD155 and CD112) are shared with CD96 and CD226, which cause co-inhibition and co-stimulation signals, respectively. TIGIT competes with CD226 for binding to CD155 and presents higher affinity to CD115 than CD226 (Figure 2).

The immunosuppressive action of TIGIT is caused by several mechanisms. Firstly, TIGIT could inhibit CD8 T lymphocyte proliferation by acting directly on the formation of the TCR complex [95]. Secondly, TIGIT could also inhibit NK cells directly and blunt their production of interferon gamma (IFNγ) [96]. Thirdly, TIGIT could block the homodimerization of CD226, thus inhibiting its lymphocyte co-stimulation signal [97]. Finally, TIGIT is expressed on Treg lymphocytes and could induce the production of IL-10, trigger Treg activation, and suppress the production of IL-12 by DCs [98]. In mouse models, blocking TIGIT individually has little effect on survival and tumor growth, but the combination of aPD-L1 and anti-TIGIT (aTIGIT) has shown complete response with prolonged survival, as well as better efficacy than aPD-(L)1 alone [97]. In humans, CD155 overexpression is associated with resistance to ICI treatment in NSCLC [99,100], and TIGIT expression is significantly overexpressed in many cancers, particularly NSCLC [101]. Importantly, mAb-targeting TIGIT, with the property of co-engaging the crystallizable fragment (Fc) with the FcγR fragment, enable an improvement in response via a reduction in tumor volume, probably because of a reduction in Treg infiltrate and an increase in infiltration of NK immune cells and macrophages within the TME, leading to increased cytokine release [79,102]. Based on such data, mAbs are in development in clinical trials. aTIGIT mAbs can be divided into two categories, namely mAbs with a so-called “silent” Fc (Fc-silent, Fcs) and “activated” mAbs (Fc-enabled or Fc-active, Fce), which could trigger antibody-dependent cytotoxicity (ADCC). Fce antibodies are able to interact with several receptors, whereas Fcs antibodies bind to none. The impact of the use of these two antibodies in combination with an aPD-1 agent on the TME and peripheral circulation has recently been demonstrated in murine models and in vitro in humans. Fcs antibodies induce reactivation of tumor-specific CD8 T lymphocytes that had undergone exhaustion, without inducing intratumor and peripheral Treg depletion. In contrast, the Fce antibody induces intratumor and peripheral Treg depletion [103]. Among the aTIGIT antibodies developed or in development, domvanalimab is currently one of the only compounds being tested in phase II and III that belongs to the Fcs family. Other aTIGIT mAbs, such as tiragolumab and vibostolimab, belong to the Fce family. Published results from clinical trials with aTIGIT mAb and bsAb are summarized in Table 2.

### 4.1. Tiragolumab

CITYSCAPE, the first large-scale trial using aTIGIT mAbs, was a phase II study evaluating the combination of tiragolumab (or placebo) with the aPD-L1 agent atezolizumab as a first-line treatment for locally advanced or mNSCLC in PD-L1-positive patients (TPS score ≥ 1). This study also investigated the impact of tumor PD-L1 as a predictive biomarker of response to the combination of tiragolumab and atezolizumab. The study showed a significant benefit in favor of the study combination on the primary endpoints (PFS and ORR) in the overall population, with results strongly influenced by PD-L1 expression, with more significant results in the TPS > 50% population. An improvement in OS was also observed in the PD-L1 50% group [104]. Tissue sample analyses from this study demonstrated that a high baseline level of intratumoral tumor-associated macrophages (TAM) and Treg cells was associated with improved OS only for patients treated with the tiragolumab and atezolizumab combination. Preclinical experiments in mouse models suggest that aTIGIT mAbs activate the myeloid compartment (TAM, monocytes, and DCs), thus reprogramming exhausted CD8 T cells to a memory-like state [105]. The phase III SKYSCRAPER-01 trial has further evaluated the combination of tiragolumab/atezolizumab versus placebo/atezolizumab, in the first-line treatment for mNSCLC with PD-L1 > 50%. Initial communications from the laboratory on the interim analyses show no benefit in terms of PFS or OS [106]. SKYSCRAPER-06 is also a randomized phase III trial that assessed this tiragolumab/atezolizumab combination associated with chemotherapy versus pembrolizumab plus chemotherapy, in the first-line setting for non-squamous mNSCLC. Recently published results showed no improvement of PFS and OS with the addition of the aTIGIT mAb to the chemotherapy and aPD-L1 combination [107].

### 4.2. Domvanalimab

Domvanalimab is an Fc-silent humanized IgG1 that targets TIGIT. The ARC-7 phase II trial (NCT04262856) is a randomized phase II study evaluating the use of zimberelimab, an aPD-1 mAb in combination with domvanalimab alone or in combination with etrumadenant (an antagonist of adenosine A2a and A2b receptors on immune cells) in treatment-naive patients with PDL1> 50% (TPS) and no targetable oncogenic addiction. The co-primary endpoints were ORR and PFS. With domvanalimab added to the aPD-1, ORR increased from 12% to 18%, and median PFS from 5.4 to 12.0 months [108]. The phase II ARC-10 study (NCT04736173) is currently evaluating the zimberelimab/domvanalimab combination versus pembrolizumab alone in locally advanced NSCLC or mNSCLC, not pretreated, with PD-L1 > 50% and without targetable oncogenic addiction. The primary endpoint is PFS with the first results provided by Arcus biosciences [109,110]. Another randomized phase III trial, STAR-121 (NCT05502237), is evaluating the dual combination of zimberelimab/domvanalimab with a platinum-based doublet versus a pembrolizumab/platinum-based doublet. The target population for this study comprises treatment-naive patients with mNSCLC without a targetable mutation and irrespective of PD-L1 expression. The primary endpoints are PFS and OS [111]. Finally, the phase II, VELOCITY-Lung study (NCT05633667) is evaluating three experimental treatment arms in patients diagnosed with mNSCLC without prior treatment or targetable mutations, irrespective of their PD-L1 status: the anti-TROP-2 conjugate antibody sacituzumab-govitecan combined with the dual immunotherapy zimberelimab/domvanalimab, a second experimental arm combining etrumadenant with the zimberelimab/domvanalimab doublet, and, finally, an etrumadenant/zimberelimab arm. The study was launched in March 2023 and is currently recruiting patients [112].

### 4.3. Vibostolimab

Vibostolimab is a human IgG1 aTIGIT mAb that was first tested in humans in a phase I trial (NCT02964013) with a pan-tumor arm A and an arm B that included patients with mNSCLC who were treatment-naïve or had been pretreated with one or more lines of treatment, including anti PD-(L)1 immunotherapy [113]. This study led to the phase II KEYVIBE-002 study [114] (NCT04725188) that evaluated the combination of pembrolizumab/vibostolimab with or without docetaxel versus standard second-line docetaxel monotherapy in patients with mNSCLC progressing after a standard first-line platinum-based doublet and aPD-(L)1 immunotherapy, with PFS as a primary endpoint. Median PFS was 5.6 months in the pembrolizumab/vibostolimab and docetaxel arm, 2.7 months in the pembrolizumab/vibostolimab arm, and 3.2 months in the docetaxel arm. Two other studies are ongoing, namely KEYVIBE-003 [115] (NCT04738487), with a pembrolizumab/vibostolimab regimen versus pembrolizumab alone in the first-line treatment for patients diagnosed with mNSCLC with tumor PD-L1 > 1% (TPS), with OS as the primary endpoint, and KEYVIBE-007 [116] (NCT05226598) with a pembrolizumab/vibostolimab regimen combined with a platinum-based doublet versus the standard first-line regimen of pembrolizumab and a platinum-based doublet for systemically naive mNSCLC, regardless of PD-L1 status, and with OS as the primary endpoint.

bsAbs that target TIGIT and other immune checkpoints have been developed in clinical trials with results for NSCLC patients.

### 4.4. Rilvegostomig (AZD2936)

Rilvegostomig is a bsAb targeting both TIGIT and PD-1 that demonstrated promising early signs of tolerability and efficacy in patients with advanced or metastatic PD-L1-positive NSCLC, following progression on at least one prior ICI (part A, B) according to initial findings from the phase 1/2 ARTEMIDE-01 study (NCT04995523) presented at the 2023 ASCO and 2023 ESMO annual meetings [117,118]. The ORR with rilvegostomig was 4.8% and was comprised entirely of partial responses (PR). The median PFS was 2.1 months, and the 6-month disease control rate (DCR) was 31.3% [118]. Rilvegostomig was well tolerated, with no dose-limiting toxicity observed and no grade 4 or 5 treatment-related adverse events (TRAEs) [117]. The ARTEMIDE-01 study also comprised cohorts of ICI-naïve, previously treated patients with mNSCLC and PD-L1 TPS > 1% (part C, D) [119]. The patients with a TPS of 1–49% had an ORR of 29.0%. The patients with a TPS > 50% were randomized between 750 mg and 1500 mg of rilvegostomig every 3 weeks, achieving ORRs of 61.8% and 36.7%, respectively. The 750 mg dose was selected based on these results, on modeling analysis, and on intratumoral receptor occupancy. In flow cytology analyses, doses of rilvegostomig of 210 mg or greater were found to sustain roughly 90% occupancy of PD-1 and TIGIT receptors on peripheral T cells. The ARTEMIDE-01 study continues to enroll participants with a cohort in the first-line setting of mNSCLC (part E). Additionally, AstraZeneca, the developer of rilvegostomig, has announced plans to initiate a phase 3 study to further explore the agent. Rilvegostomig is also being explored in various combinations for gastric cancer (NCT05702229) and in other lung cancer settings (NCT04612751).

**Table 2:** Published data from clinical trials with anti-TIGIT monoclonal antibodies or bispecific antibodies in non-small-cell lung cancer.

aTIGIT mAb	Type	Study	Phase	Setting	N	Intervention	1st Endpoint	Results with aTIGIT	Ref
**Tiragolumab**	Human IgG1κ	CITYSCAPE	II	Metastatic, 1st line, PD-L1 ≥ 1%	135	Tirago + atezo (aPD-L1) vs. pcb + atezo	ORR, PFS	ORR 31·3% with atezo + tirago vs. 16·2% with atezo + pcb (*p* = 0·031) mPFS 5·4 mo with atezo + tirago vs. 3·6 mo with atezo + pcb (HR 0·57, *p* = 0·015) Serious TRAE 21% with tirago + atezo vs. 18% with atezo + pcb	[104]
		SKYSCRAPER-01	III	Metastatic, 1st line, PD-L1 > 50%	534	Tirago + atezo vs. pcb + atezo	PFS, OS	mOS 22.9 mo with atezo + tirago vs. 16.7 mo with atezo + pcb (HR, 0.81; 95% CI, 0.63–1.03) No safety data	[106]
		SKYSCRAPER-06	III	Metastatic, 1st line	542	Tirago + atezo + CT vs. pembro + CT	PFS, OS	mOS 18.9 mo with tirago + atezo + CT vs. 23.1 mo with pembro + CT (HR, 1.3; 95% CI, 1.0–1.7) mPFS 8.3 mo with tirago + atezo + CT vs. 9.9 mo with pembro + CT (HR, 1.3; 95% CI, 1.0–1.6) Grade ≥ 3 TRAE 61% with tirago + atezo + CT vs. 61% with pembro + CT	[107]
**Domvanalimab**	Fc-silent humanized IgG1	ARC-7	II	Metastatic, 1st line, PD-L1 ≥50%	151	Domva + zimbe (aPD-1) + etrumadenant vs. domva + zimbe vs. zimbe	ORR, PFS	ORR 18% with domva + zimbe vs. 12% with zimbe mPFS 12 mo with domva + zimbe vs. 5.4 mo with zimbe (HR 0.55, 95% CI 0.31–1.0) Grade ≥ 3 TEAE 47% with domva + zimbe vs. 58% with zimbe	[108]
		ARC-10	II	Metastatic, 1st line, PD-L1 ≥50%	98	Domva + zimbe vs. zimbe vs. CT	PFS	mPFS 11.5 mo with domva + zimbe vs. 6.5 mo with zimbe (HR 0.69, 95% CI 0.40–1.18) mOS NR with domva + zimbe vs. 24.4 mo with zimbe (HR 0.64, 95% CI 0.32–1.25) Grade ≥ 3 TRAE 21.1% with domva + zimbe vs. 15.0% with zimbe	[109,110]
**Vibostolimab**	Human IgG1	KEYVIBE-002	II	Metastatic, 2nd line	225	MK-7684A (vibo + pembro [aPD-1]) + docetaxel vsMK-7684A (vibo + pembro) vs. pcb + docetaxel	PFS	mPFS (95% CI) was 5.6 mo with vibo + pembro + docetaxel, 2.7 mo with vibo + pembro vs. 3.2 mo with pcb + docetaxel HR 0.77 (95% CI 0.53–1.13; *p* = 0.0910) for vibo + pembro + docetaxel vs. pcb + docetaxel; HR 1.40 (0.96–2.02), *p* = 0.9622 for vibo + pembro vs. pcb + docetaxel TRAE of any grade 96.5% with vibo + pembro + docetaxel vs. 60.3% with vibo + pembro	[114]
**aTIGIT bsAb**	**Type**	**Study**	**Phase**	**Setting**	**N**	**Intervention**	**1st Endpoint**	**Results with aTIGIT Bispecific**	**Reference**
**Rilvegostomig**	TIGIT/PD-1	ARTEMIDE-01	I/II	Metastatic, PD-L1 ≥ 1%, 2nd line, previous ICI (part A, B)	83	Rilve 750 or 1500 mg	Safety	ORR = 4.8% DCR = 31.3% mPFS = 2.1 m Grade ≥ 3 TRAE 8.4%	[117,118]
				Metastatic, PD-L1 ≥ 1%, ≥2nd line, ICI-naïve (part C, D)	96	Rilve 750 or 1500 mg	ORR	ORR PD-L1 1–49% with rilve 750 mg: 29% ORR PD-L1 ≥ 50% with rilve 750 mg: 61.8% ORR PD-L1 ≥ 50% with rilve 1500 mg: 36.7% Grade ≥ 3 TRAE 10.5%	[119]

mAb: monoclonal antibody; N: number of patients included; tirago: tiragolumab; atezo: atezolizumab; pcb: placebo; pembro: pembrolizumab; ORR: objective response rate; PFS: progression-free survival; mPFS: median progression-free survival; mo: months; HR: hazard ratio; OS: overall survival; mOS: median overall survival; CI: confidence interval; TRAE: treatment-related adverse events; domva: domvanalimab; zimbe: zimberelimab; CT: chemotherapy; TEAE: treatment-emergent adverse events; NR: not reached; vibo: vibostolimab; pembro: pembrolizumab; bsAb: bispecific antibody; ICI: immune checkpoint inhibitor; rilve: rilvegostomig; DCR: disease control rate.

## 5. LAG-3 Targeting

**Figure 3:**
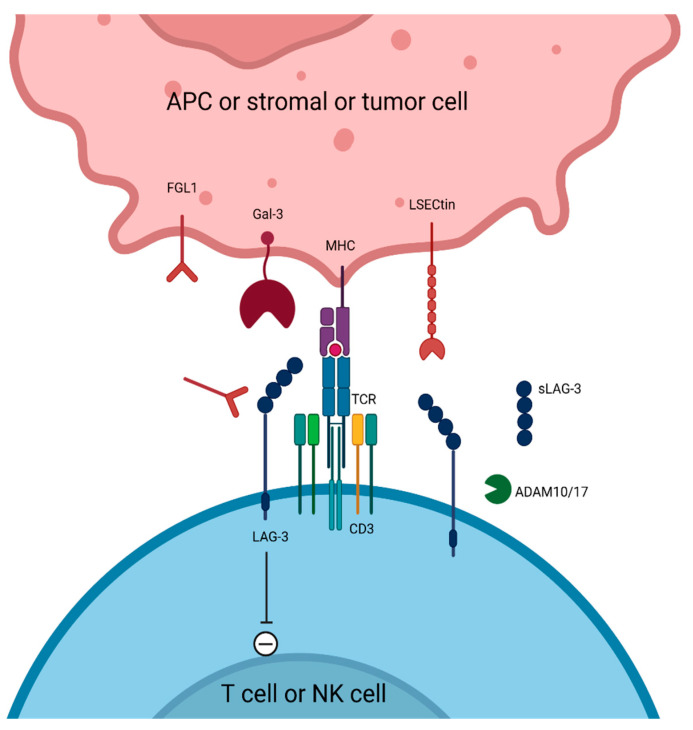
LAG-3 mechanism of action. APC: antigen-presenting cell; FGL1: fibrogen-like protein 1; Gal-3: galectin-3; sLAG-3: soluble LAG-3.

Lymphocyte activation gene-3 (LAG-3, reviewed in references [120,121,122]), also known as CD223, is a type I transmembrane protein located on human activated CD4 T cells, CD8 T cells, NK cells, gamma/delta T cells, and plasmacytoid DCs, as well as on rested/activated Tregs. Although LAG-3 and CD4 have a homologous structure, the two receptors share only 20% of their amino acid sequence. Singularly, LAG-3 is a non-ITIM IR that functions through critical “KIEELE” and “EP” motifs on the cytoplasmic tail [123]. LAG-3 on immune T cell lines or NK cells binds to several ligands, among which class II major histocompatibility complex (MHC-II) is the canonical one. Additionally, in the cancer context, LAG-3 also binds fibrogen-like protein 1 (FGL1) released by cancer cells [124], galectin-3 on tumor and tumor-associated stroma cells [125], and LSECtin on tumor cells [126]. Mechanistically, LAG-3 acts as an IR through several immunosuppressive functions (Figure 3). Firstly, it impairs the functionality of immunocompetent adaptive cells. LAG-3 may constitutionally associate with the TCR-CD3 complex in CD4 and CD8 T cells, independently of the presence of MHC-II, although it is the canonical LAG-3 ligand. After TCR stimulation, LAG-3 and TCR colocalization rapidly increases within the immune synapse, further limiting CD4 and CD8 T cell activation and expansion. Mechanistically, the LAG-3 cytoplasmic tail disrupts LcK-CD4 or LcK-CD8 interaction, thereby inhibiting TCR signaling [123,127]. Beyond the membrane receptor structure, LAG-3 is shed at the cell surface in a soluble form (sLAG-3) by ADAM10 on rested/activated T cells, and ADAM17 on activated T cells only. This sLAG-3 release through metalloproteinase cleavage promotes an antitumor immune response and is required for T cell function [128].

Secondly, it impairs innate immune cell functions such as APC. Conversely, LAG-3-expressing Tregs interact with APC to mediate “reverse” signaling that does not require the LAG-3 cytoplasmic tail. Rather, this LAG-3-mediated DC inhibition relies on cell contact with LAG-3-MHC-II interaction, subsequent ERK activation, and SHP1 recruitment [129]. Thirdly, LAG-3 supports immunosuppressive cell functions. LAG-3 expression is critical for Treg suppressor activity [129,130,131,132]. Mechanistically, LAG-3 mediates PI3K-MTORC-AKT inhibition in Foxp3+ Tregs, thus reducing Myc expression and regulating Treg metabolism that allows normal suppressive function [133]. Especially in NSCLC, a transcriptomic study of lung tumor-infiltrating Tregs obtained from patients revealed divergent Treg clusters, among which highly immunosuppressive “activated Tregs” had markedly increased expression of LAG-3 [134].

As described above, the expression of LAG-3 is a well-known marker of T cell exhaustion. In order to impair LAG-3 immunosuppressive functions, LAG-3-targeted mAbs have been designed. Published results from clinical trials with aLAG-3 mAbs and bsAbs are summarized in Table 3.

### 5.1. Relatlimab (BMS-986016)

Relatlimab is a human IgG4 mAb; it was the first LAG-3 inhibitor developed [135] and was first approved for the treatment of melanoma. In early-stage NSCLC, the randomized phase II NEOPredict trial compared short-course neoadjuvant nivolumab versus short-course neoadjuvant nivolumab + relatilmab association in resectable stage IB–IIA [45,136] (single-level N2, UICC 8th edition). In the experimental arm, 50% of patients had adenocarcinoma, and 30% had squamous cell lung cancer. Stage I, IIA-B, and IIIA represented 33.3%, 56.7%, and 10% of this arm, respectively. Overall, 73.3% had PD-L1-positive disease. The primary endpoint was met, since all patients underwent surgery within 43 days after treatment initiation, in both arms. Regarding secondary outcomes, a higher ORR was observed with relatilmab addition (27% vs. 10%), but no radiological complete response occurred. Pathological complete response was increased from 13 to 17% with relatilmab, as were the rates of major pathological responses (≤10% viable tumor cells; MPR) (from 27 to 30%), while pathological response rates (≤50% viable tumor cells) increased from 60 to 72%. Having PD-L1-positive disease was associated with better pathological response. There was no statistical difference in 12-month DFS or OS, which were excellent in both arms [45]. Immune cell phenotyping revealed that responders had a significant increase in peripheral blood CD8+ and GZM + CD8+ and lower myeloid (neutrophils, monocytes) and Treg tumor infiltration. Regarding gene expression, nivolumab monotherapy induced LAG-3 gene expression, while the combination arm did not. In mNSCLC, the combination of relatlimab, nivolumab, and platinum-based chemotherapy was evaluated in the first-line setting in the phase II RELATIVITY-104 trial. The use of relatlimab led to an increase in ORR from 43.7% with nivolumab–chemotherapy to 51.3% with the addition of LAG-3 mAbs. This benefit was observed in particular in a subgroup of patients with PD-L1 >= 1 and non-squamous histology disease, with an ORR of 58.0 vs. 39.6% in the arm without relatlimab [137].

### 5.2. Ieramilimab (LAG-525)

Ieramilimab is a humanized IgG4 mAb that contains the S228 hinge-stabilizing mutation, thus blocking the LAG-3–MHC-II interaction with low nanomolar affinity. After having demonstrated safety as a monotherapy or in combination with an aPD-1 in a phase I study [138], ieramilimab was further tested in a phase II study conducted in advanced solid tumors, in which 42 mNSCLC PD-L1-unselected patients were included [139]. While the ORR was 15% among the pretreated ICI-naïve patients, there were no responders among the patients pretreated with aPD-1/PD-L1. The median PFS was 4.2 and 3.9 months for ICI-naive vs. ICI-pretreated NSCLC patients, respectively. The responding patients had a higher T cell-inflamed signature and LAG-3 expression at baseline than the non-responders.

### 5.3. Favezelimab (MK-4280)

Favezelimab [140] is a humanized IgG4 LAG-3-targeted mAb that binds the interacting site with MHC class II. The Keynote 495/KeyIMPACT [141,142] trial is a biomarker-directed adaptively randomized multi-cohort phase II study in the first-line setting of mNSCLC. Patients were categorized by the 18 immune-related gene signatures that define the T cell-inflamed gene expression profile (TcellinfGEP) [24,25,26] and TMB [22,23] before randomization. One of the study arms consisted of pembrolizumab + favezelimab 200 mg, which was then changed for favezelimab 800 mg. Patient categorization consisted of a TcellinfGEPlowTMBnon-high (group I), TcellinfGEPlowTMBhigh (group II), TcellinfGEPnon-lowTMBnon-high (group III), and TcellinfGEPnon-lowTMBhigh (group IV). With pembrolizumab + favezelimab 800 mg, the ORRs were 27.3% in group 2, 13.6% in group 3, and 50% in group 4. The corresponding mPFS was 4.2, 3.5, and 13 months, respectively, while the corresponding mOS was 24.5, 11 months, and not reached, respectively. When the ORR was evaluated by TMB status alone in this arm, the response rates ranged from 14% in patients with TMBnon-high tumors to 41% in patients with TMBhigh. When the ORR was evaluated by PD-L1 status, the response rates ranged from 17% in patients with TPS < 1% tumors, 42% in patients with TPS ≥ 1%, 33% in patients with TPS 1–49% tumors, and 55% in patients with TPS ≥ 50% tumors. The ORR by dual TMB and PD-L1 status ranged from 11% in patients with PD-L1 TPS < 1% and TMBnon-high tumors to 64% in patients with PD-L1 TPS ≥ 1% and TMBhigh tumors. However, the combination of pembrolizumab + favezelimab did not pass the response rate prespecified efficacy threshold in any dual biomarker-based subgroup in this study.

### 5.4. Fianlimab (REGN3767)

Fianlimab is a humanized IgG4 antibody mAb that has been evaluated in the first-line setting of mNSCLC. For patients with PD-L1 >= 50%, a three-arm, randomized, phase II/III study (NCT05785767) is assessing fianlimab high dose + cemiplimab (aPD-1), fianlimab low dose + cemiplimab, and placebo + cemiplimab, with ORR and OS as primary endpoints in the phase II and phase III parts, respectively [143]. For PD-L1-unselected patients, a three-arm, phase II/III study (NCT05800015) is ongoing [144]. The phase III part consists of fianlimab high or low dose (according to the dose determined in the phase II) + cemiplimab + platinum-doublet chemotherapy vs. placebo + cemiplimab + chemotherapy. In early-stage NSCLC (stage II-IIIB), a randomized phase II peri-operative study (NCT06161441) is testing the addition of fianlimab on top of a combination of aPD-1 + platinum-doublet chemotherapy.

Other LAG-3-targeted mAbs are currently under development in early phase trials, but safety and efficacy data in NSCLC patients are not yet available. These include miptenalimab (BI 754111) [145,146,147], a humanized IgG4 anti-LAG-3 mAb (NCT03780725, NCT03964233, NCT03156114, NCT03697304, NCT03433898), TSR 033 (NCT03250832) [148], a humanized IgG4 anti-LAG-3 mAb; Sym022 [149], a Fc-inert human monoclonal antibody targeting LAG-3 (NCT04641871, NCT03311412, NCT03489369), and Tuparstobart (ICAGN02385), a humanized Fc-engineered IgG1κ (NCT03538028).

Beyond mAbs, bsAbs targeting both LAG-3 and PD-(L)1 have been engineered, such as FS118 (LAG-3/PD-L1) [150], tomebstomig RO7247769 (LAG-3/PD-1) [151], and tebotelimab MGD013 (LAG-3/PD-1) [152,153]. All these drugs have been evaluated in phase I basket trials comprising mNSCLC, with a low number of patients and with safety as a primary endpoint. The ORRs ranged from 0% to 17.4% in these studies and probably had less efficacy in the ICI-pretreated mNSCLC patients.

**Table 3:** Published data from clinical trials with anti-LAG-3 monoclonal antibodies or bispecific antibodies in non-small-cell lung cancer.

aLAG-3 mAb	Type	Study	Phase	Setting	N	Intervention	1st Endpoint	Results with aLAG-3	Ref
**Relatlimab** **(BMS-986016)**	Human IgG4	NEOPredict	II	Early, resectable, stage IB–IIA (single-level N2, UICC 8th edition)	60	Nivo (aPD-1) + relavs. nivo	Feasibility of surgery within 43 days	Surgery within 43 days: 100% ORR 27% with nivo + rela % vs. 10% with nivo pCR 17% with nivo + rela % vs. 13% with nivo MPR 30% with nivo + rela % vs. 27% with nivo PR 72% with nivo + rela % vs. 60% with nivo Grade ≥ 3 AE 53% with nivo + rela vs. 40% with nivo	[45]
		RELATIVITY-104	II	Metastatic, 1st line	309	Nivo + rela + CT vs. nivo + CT	ORR	ORR 51.3% with nvo + rela + CT vs. 43.7% with nivo + CT mDoR 10.1 mo with nvo + rela + CT vs. 9.1 mo with nivo + CT Grade ≥ 3 TRAE 54% with nivo + rela + CT vs. 55% with nivo + CT	[137]
**Ieramilimab** **(LAG-525)**	Humanized IgG4	Lin et al.	II	Metastatic, ≥2nd line	42 with NSCLC	Iera + sparta (aPD-1)	ORR	ORR 15% mPFS 4.2 mo for ICI-naive and 3.9 months for ICI-pretreated patients Serious AE 8.5% in ICI-naïve vs. 2.2% in ICI-pretreated patients	[139]
**Favezelimab** **(MK-4280)**	Humanized IgG4	KEYNOTE-495/KeyImPaCT	II	Metastatic, 1st line	243	Pembro (aPD-1) +lenvatinib vs. pembro + quavon (aCTLA-4) vs. pembro + fave 800 mg	ORR	ORR 29.4% ORR 27.3% in Tcell_inf_GEP^low^TMB^high^, 13.6% in Tcell_inf_GEP^non-low^TMB^non-high^, 50% in Tcell_inf_GEP^non-low^TMB^high^. mPFS 4.2 mo in Tcell_inf_GEP^low^TMB^high^, 3.5 mo in Tcell_inf_GEP^non-low^TMB^non-high^, 13 mo in Tcell_inf_GEP^non-low^TMB^high^. mOS 24.5 mo in Tcell_inf_GEP^low^TMB^high^, 11 mo in Tcell_inf_GEP^non-low^TMB^non-high^, NR in Tcell_inf_GEP^non-low^TMB^high^. Grade ≥ 3 TRAE with 68.8% pembro + lenvatinib vs. 23.2% with pembro + quavon vs. 21.6% with pembro + fave 800 mg	[141,142]
**aLAG-3 bsAb**	**Type**	**Study**	**Phase**	**Setting**	**N**	**Intervention**	**1st endpoint**	**Results with aLAG-3**	**Ref**
**FS118**	LAG-3/PD-L1	Yap et al.	I	Metastatic, ≥2nd line	9 with NSCLC	FS118	Safety	ORR = 0% DCR = 33.3% Grade ≥ 3 TRAE 4.7%	[150]
**Tebotelimab (MGD013)**	LAG-3-/PD-1	Luke et al.	I	Metastatic, ≥2nd line	35 with NSCLC	Tebo	Safety	ORR = 14% in ICI-naïve ORR = 0% in ICI-pretreated Grade ≥ 3 TRAE 22.3%	[152,153]

mAb: monoclonal antibody; N: number of patients included; nivo: nivolumab; rela: relatlimab; ORR: objective response rate; pCR: pathological complete response; MPR: major pathological response; PR: pathological response; AE: adverse events; TRAE: treatment-related adverse events; CT: chemotherapy; mDoR: median duration of response; mo: months; iera: ieramilimab; sparta: spartalizumab; mPFS: median progression-free survival; pembro: pembrolizumab; quavon: quavonlimab; fave: favezelimab; TMB: tumor mutational burden; bsAb: bispecific antibody; DCR: disease control rate; ICI: immune checkpoint inhibitor.

## 6. TIM-3 Targeting

T cell immunoglobulin and mucin domain-containing protein 3 (TIM-3, reviewed in references [154,155,156]) is a type I transmembrane immunoregulatory protein that belongs to the TIM family. TIM-3 is located on human activated CD4 T cells, CD8 T cells [157], NK cells [158], Tregs [159], myeloid cells [160] (such as DCs, intratumoral monocytes, and macrophages [161]), and also mast cells [162]. The immunoglobulin variable (IgV)-like domain and the mucin domain compose the TIM-3 extracellular domain, which binds cognate ligands. The TIM-3 cytoplasmic tail contains Y256 and Y263 (Y265 and Y272 in humans) residues that interact with HLA-B-associated transcript 3 (BAT3) and the Src family tyrosine kinases LCK and FYN. TIM-3 on immune T cell lines, myeloid cells, and NK cells binds to several ligands: galectin-9 [163,164,165,166] and CEACAM1 on tumor cells/T cell lines/NK cells/myeloid cells (DCs, monocytes, and macrophages) [167,168,169], phosphatidylserine [170] on apoptotic cells, and extracellular high-mobility group box 1 (HMGB1) [171].

**Figure 4:**
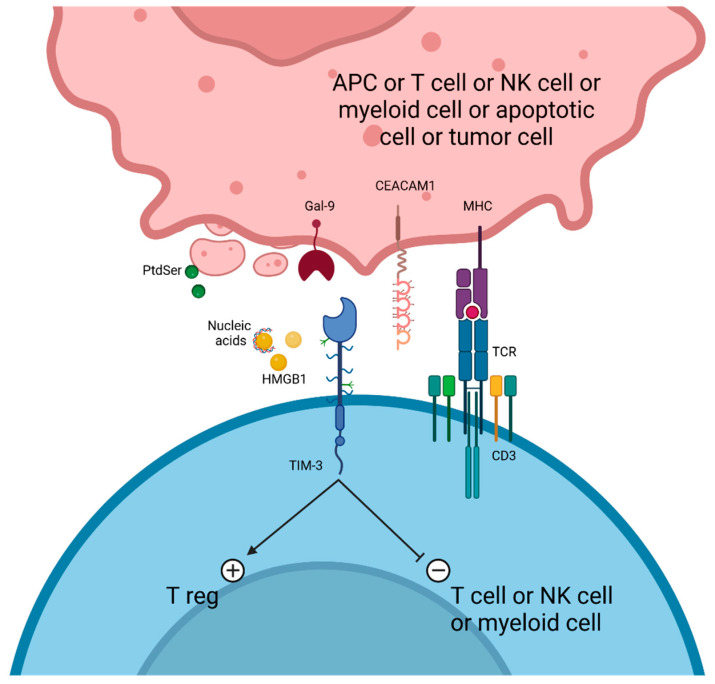
TIM-3 mechanism of action. APC: antigen-presenting cell; Gal-9: galectin-9; PtdSer: phosphatidylserine.

TIM-3 acts as an IR through several mechanisms (Figure 4): firstly by impairing functionality of immunocompetent adaptive cells. BAT3 is an adaptor protein located on the TIM-3 cytoplasmic tail that negatively regulates TIM-3 in the absence of binding ligands [172]. Galectin-9 or CEACAM1 binding on TIM-3 T cells induces phosphorylation of Y256 and Y263 residues [167,173], which releases BAT3, thus allowing TIM-3 to exert its inhibitory function. Moreover, the Src-related kinase FYN may participate in TIM-3-mediated suppressive signaling, since it binds the cytoplasmic tail and induces T cell anergy [174,175]. Galectin-9 ligation to TIM-3 can also recruit CD45 and CD148 phosphatases and disrupt the immunological synapse to further inhibit T cell functionality [176]. Secondly, TIM-3 acts by impairing innate immune cell functions, such as APC. HGMB1 mediates danger signaling through ligation on several membrane receptors, such as Toll-like receptor 2 (TLR2), TLR4, and RAGE. In particular, HMGB1 picks up nucleic acid, binds to RAGE, and triggers immunostimulatory functions through the TLR7/9 endosomal pathway [177]. TIM-3 on DCs acts as a molecular sink to sequester HMGB1 and prevents DNA capture by the alarmin [171], thus impairing DNA sensing, notably though the cGAS/STING pathway [178]. TIM-3 on DCs also negatively regulates inflammasome activation, thus decreasing the maintenance of CD8+ effector and stem-like T cells [156]. TIM-3 on DCs links with apoptotic material through phosphatidylserine and may be important for cross-presentation to CD8 T cells, a critical mechanism for ensuring peripheral tolerance [179,180,181]. Thirdly, TIM-3 acts by strengthening immunosuppressive cells. In NSCLC, a majority of intratumoral TIM-3+ CD4+ are Tregs [159,182]. Moreover, TIM-3+ Tregs are highly suppressive and produce immunosuppressive cytokines, such as IL-10 [183,184].

Regarding exhaustion, TIM-3 T cells are considered as terminally differentiated exhausted T cells [185]. Notably, expression of the transcription factor TCF1, which is critical for generation and maintenance of the pool of progenitor CD8 T cells, varies in the opposite manner to TIM-3. Indeed, progenitors of exhausted CD8 T cells are TCF1+ TIM-3-, while the terminally exhausted subset is characterized by high expression of inhibitory molecules (TIM-3+ PD-1) [34,186,187,188]. Published results from clinical trials with aTIM-3 mAbs and bsAbs are summarized in Table 4.

### 6.1. Cobolimab (TSR-022/GSK4069889)

Cobolimab is a humanized IgG4 TIM-3-targeted mAb. The phase I basket trial AMBER [189,190] included ICI pre-treated mNSCLC patients. In part 2B, the combination of cobolimab + dostarlimab (aPD-1) demonstrated modest efficacy with an ORR of 8.3% and a DCR of 21.4%. The recommended phase 2 dose (RP2D) for cobolimab was 300 mg, which was the dose with the highest ORR (9.8%) [191]. The randomized, three-arm, phase II/III COSTAR study (NCT04655976) is now evaluating dostarlimab ± cobolimab + docetaxel vs docetaxel as the control arm [191].

### 6.2. Sabatolimab MGB453

Sabatolimab is a humanized IgG4 TIM-3-targeted mAb. In a phase II study, 17 patients with mNSCLC previously treated with an aPD-(L)1 received a combination of MGB453 + spartalizumab (aPD-1) with 41.2% stable disease (SD) [192].

### 6.3. LY3321367

LY332136 is a humanized IgG1λ, Fc-null TIM-3-targeted mAb, tested as a monotherapy or in combination with the aPD-L1 LY300054 in advanced solid tumors in a phase Ia/b trial. NSCLC patients who had progressive disease as the best immunotherapy response in previous lines had worse outcomes (ORR 0%, DCR 34.8%, mPFS 1.9 months) than those who had disease control or response to prior immunotherapy (ORR 7%, DCR 50%, mPFS 7.3 months) [193]. This molecule is currently under investigation in several trials (NCT03099109, NCT02791334).

Other TIM-3-targeted mAbs are currently under development in early phase trials, but safety and efficacy data in NSCLC patients are not yet available. These include sym-023/S095018, an IgG1, with a variant engineered to remove Fc gammaR binding (NCT03311412, NCT03489343, NCT04641871) BMS-986258, a silent IgG1 (NCT03446040), INCAGN02390 [194], a fully human Fc-engineered-silent IgGk1 (NCT03652077, NCT04370704, NCT05287113), and surzebiclimab/BGB-A425, a humanized IgG1 (NCT03744468, NCT03744468).

In addition, bsAbs targeting both TIM-3 and PD-(L)1 have also been evaluated: sabestomig/AZD7789 (TIM-3/PD-1) [195] and LY-3415244 (TIM-3/PD-L1) [196]. Both bsAbs have been evaluated in early phase trials with mNSCLC, with safety as a primary endpoint. The ORR ranges from 0% to 10.5% in these studies. No results have been communicated for lomvastomig/R07121661 (TIM-3/PD-1) as yet (NCT03708328).

**Table 4:** Published data from clinical trials with anti-TIM-3 monoclonal or bispecific antibodies in non-small-cell lung cancer.

aTIM-3 mAb	Type	Study	Phase	Setting	N	Intervention	1st Endpoint	Results with aTIM-3	Ref
**Cobolimab (TSR-022/GSK4069889)**	Humanized IgG4	AMBER	I	Metastatic, ≥2nd line after aPD-1/PD-L1	84	Cobo + dostar (aPD-1)	ORR	ORR 8.3% DCR 21.4% Grade ≥ 3 TRAE 13.1%	[189,190]
**Sabatolimab (MGB453)**	Humanized IgG4	Mach et al.	I	Metastatic, ≥2nd line after aPD-1/PD-L1	17 with NSCLC	Saba + sparta (aPD-1)	ORR	SD 41.2% Grade ≥ 3 TRAE 11.8%	[192]
**LY3321367**	Humanized IgG1λ, Fc-null	Harding et al.	I	Metastatic, ≥2nd line	65 with NSCLC	LY3321367 +/−LY300054 (aPD-L1)	Safety, RP2D	PD as the best immunotherapy response in previous lines: ORR 0%, DCR 34.8%, mPFS 1.9 mo SD or PR as the best immunotherapy response in previous lines: ORR 7%, DCR 50%, mPFS 7.3 mo Grade ≥ 3 TRAE 3%with LY3321367 monotherapy	[193]
**aTIM-3 bsAb**	**Type**	**Study**	**Phase**	**Setting**	**N**	**Intervention**	**1st Endpoint**	**Results with aLAG-3**	**Ref**
**Sabestomig (AZD7789)**	TIM-3-/PD-1	Besse et al.	I/IIa	Metastatic, ≥2nd line, ICI-pretreated	39	Sabe	Safety	ORR = 10.5% DCR = 36.8% Grade ≥ 3 TRAE 0%, TRAE of any grade 41%	[195]
**LY3415244**	TIM-3/PD-L1	J1C-MC-JZDA	I	Metastatic, ≥2nd line	2 with NSCLC	LY3415244	Safety, RP2D	ORR = 0% Serious AE 16.7%	[196]

mAb: monoclonal antibody; N: number of patients included; cobo: cobolimab; dostar: dostarlimab; ORR: objective response rate; DCR: disease control rate; TRAE: treatment-related adverse events; saba: sabatolimab; sparta: spartalizumab; SD: stable disease; RP2D: recommended phase 2 dose; PD: progressive disease; mPFS: median progression-free survival; mo: months; PR: partial response; bsAb: bispecific antibody; sabe: sabestomig; AE: adverse events.

## 7. NKG2A Targeting

NKG2A (reviewed in reference [197]) is a transmembrane immunoregulatory protein that belongs to the NKG2 family (C-type lectins) [198]. NKG2A is expressed on NK, NKT, CD8 T, and γδ T cells, dimerizes with CD94, and has for a cognate ligand the non-classical class I major histocompatibility complex (MHC-I) molecules of the human leukocyte antigen (HLA)-E. The NKG2A cytoplasmic tail contains two immunoreceptor tyrosine-based inhibitory motifs, making this receptor an ITIM receptor [199,200,201].

**Figure 5:**
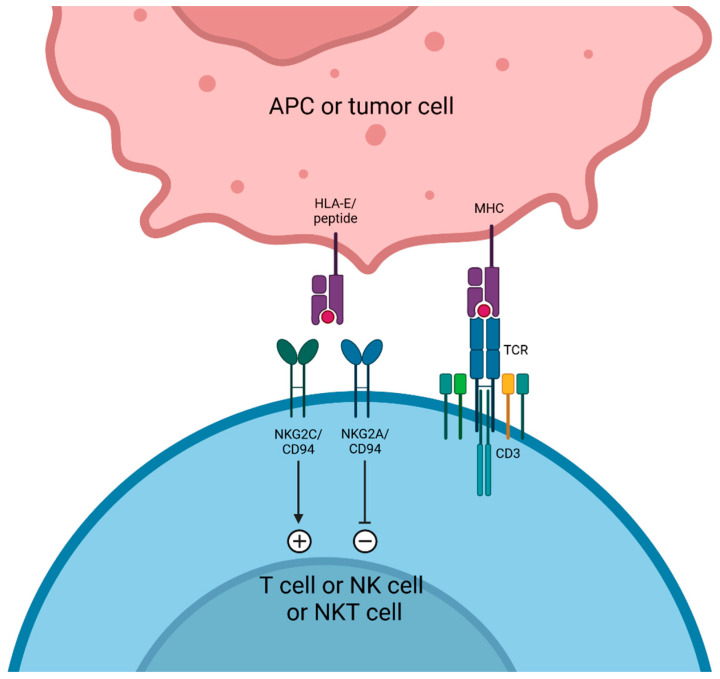
NKG2A mechanism of action. APC: antigen-presenting cell.

NKG2A acts as an IR through several mechanisms [202,203,204] (Figure 5). Firstly, by impairing functionality of immunocompetent adaptive cells: on NKG2A+ activated CD8 T cells, the ligation NKG2A-HLA-E axis induces ITIM domain phosphorylation, which recruits the SHP-1 and SHP-2 tyrosine phosphatases [205,206,207,208]. Chronologically, the upregulation of NKG2A by CD8 T cells seems to occur after repeated antigen stimulation. NKG2A may be considered as a later immune checkpoint than PD-1, LAG-3, and TIGIT [209]. Secondly, by impairing innate immune cell functions: as described above, on NKG2A+ NK cells, HLA-E binding protects target cells from lysis [210]. Notably, on NK cells, HLA-E can also bind to the CD94/NKG2C heterodimer. Contrary to NKG2A, NKG2C is an ITAM receptor that cooperates with DAP12 to trigger an activating signal [211]. However, the binding affinity of CD94/NKG2A is approximatively six times higher than CD94/NKG2C [212,213]. The balance between the signaling of these two contradictory receptors may also depend on the type of HLA-E-presented peptide on the target cell [214,215,216]. Thirdly, by modulating the TME: NK cells might influence the polarization of immune cells in the TME through its NKG2 receptors. Indeed, activation of NK cells may trigger a TAM switch by favoring differentiation of a TAM1 subset [217]. Together, these data suggest that targeting the CD94/NKG2A-HLA-E axis may enhance immunotherapy efficacy. Published results from clinical trials with aNKG2A mAbs are summarized in Table 5.

### Monalizumab

Monalizumab is a humanized IgG4-blocking mAb specific to NKG2A (it does not bind NKG2C) [218]. In the neoadjuvant setting, among 83 patients enrolled in the phase II NeoCOAST [219] platform trial, 20 received a single cycle of monalizumab with durvalumab before surgery for stage IA3-IIIA NSCLC. With monalizumab added to aPD-L1, the rate of major pathologic response (MPR) increased from 11.1% to 30.0%, while the pCR rate increased from 3.7% to 10%. Interestingly, the density of NKG2A+ cells in the tumor area before treatment was not associated with MPR. RNA sequencing of both pre- and post-treatment specimens showed that NK- and CD8 T cell-associated gene expression was increased in the combination arm. In a more advanced setting, the phase II open-label randomized COAST study [220,221] enrolled unresectable stage III NSCLC patients and assessed the addition of monalizumab or oleclumab (aCD73 mAb) to adjuvant durvalumab after chemoradiation. Monalizumab in addition to durvalumab increased ORR from 23.9% with durvalumab monotherapy to 40.3%, and prolonged mPFS to 19.8 months, versus 7.3 months with durvalumab monotherapy. The combination might also prolong OS (data not mature). On the basis of these results, the combination of monalizumab or oleclumab with durvalumab is currently being evaluated in the double-blind, randomized, placebo-controlled PACIFIC-9 study [222]. In the metastatic setting, the monalizumab and durvalumab combination was assessed in a phase I/II basket study with 20 previously treated but immunotherapy-naïve mNSCLC patients. In this population, aNKG2A with durvalumab showed modest efficacy, with ORR, mPFS, and mOS reaching 10%, 1.9 months, and 8.8 months, respectively. Regarding immune activation in the periphery, the addition of monalizumab increased blood levels of CXCR3-related chemokines (CXCL10 and CXCL11). In the tumor, although there was an increased number of proliferative T (CD3+ Ki67+) and cytotoxic cells (GZMB+) with the combination, no significant difference was observed in NK (Nkp46+) cells [223]. The phase Ib/IIA PIONeeR study [224] tested similar durvalumab and monalizumab therapy, but in mNSCLC patients who had already experienced disease progression after sequential or concomitant PD-(L)1 and platinum-based chemotherapy. The 12-week DCR reached with the combination was 24.1% vs. 54.5% with docetaxel, thus suggesting lower efficacy in this context. Many studies of combinations of other immunotherapies with monalizumab are currently enrolling patients, both in a curative and palliative setting (NCT06152523, NCT03801902: ARCHON-1 Trial, NCT05221840, NCT03794544, NCT05061550).

Other NKG2A-targeted mAbs are currently under development in first-line mNSCLC phase II trials: S095029, a human Fc-attenuated IgG1 mAb that binds NKG2A with an nM-range affinity [225,226] (NCT06162572), and BMS-986315, a human aNKG2A mAb (NCT06094296).

**Table 5:** Published data from clinical trials with anti-NKG2a monoclonal antibodies in non-small-cell lung cancer.

aNKG2A mAb	Type	Study	Phase	Setting	N	Intervention	1st Endpoint	Results with aLAG-3	Ref
**Monalizumab**	Humanized IgG4	NeoCOAST	II	Resectable, stage IA3-IIIA, neoadjuvant	83	Mona + durva (aPD-1) vs. ole (aCD73) + durva vs. danvatirsen + durva vs. durva	MPR	MPR 30.0% with mona + durva vs. 11.1% with durva pCR 10.0% with mona + durva vs. 3.7% with durva Grade ≥ 3 TRAE 0% with mona + durva and 0% with durva	[219]
		COAST	II	Unresectable, stage III, after chemoradiation	189	Mona + durva vs. ole + durva vs. durva	ORR	ORR 40.3% with mona + durva, vs. 23.9% with durva mPFS 19.8 mo with mona + durva vs. 7.3 mo with durva (HR 0.63, 95% CI 0.40, 0.99) mOS NR with mona + durva vs. 40.9 mo with durva (HR 0.77, 95% CI 0.44, 1.33) Grade ≥ 3 TEAE 32.8% with mona + durva vs. 34.8% with durva	[220,221]
		Patel et al.	I/II	Metastatic, 2nd or 3rd line, ICI-naive	20 with NSCLC	Mona + durva	Safety	ORR 10% mPFS 1.9 mo mOS 8.8 mo Grade ≥ 3 TRAE 20% for NSCLC patients	[223]
		PIONeeR	Ib/IIA	Metastatic, 2nd or 3rd line, after aPD-(L)1	114	Mona + durva vs. ole + durva vs. ceralasertib + durva vs. savolitinib + durva vs. docetaxel	12 w DCR	12 w DCR 24.1% with mona + durva vs. 54.5% with docetaxel No safety data	[224]

mAb: monoclonal antibody; N: number of patients included; mona: monalizumab; durva: durvalumab; ole: oleclumab; MPR: major pathological response; pCR: pathological complete response; TRAE: treatment-related adverse events; ORR: objective response rate; mPFS: median progression-free survival; mo: months; HR: hazard ratio; CI: confidence index; mOS: median overall survival; TEAE: treatment-emergent adverse events; ICI: immune checkpoint inhibitor; 12 w DCR: 12-week disease control rate.

## 8. Targeting the CD39/CD73/Adenosine Pathway

CD39 (ENTPD1) and CD73 (ecto-5′-nucleotidase) are key enzymes in the extracellular adenosine pathway and play a critical role in modulating the TME (reviewed in references [227,228]). CD39 hydrolyzes adenosine triphosphate (ATP) and adenosine diphosphate (ADP) into adenosine monophosphate (AMP), while CD73 subsequently converts AMP to adenosine (ADO). Adenosine accumulation in the TME leads to potent immunosuppressive effects, aiding tumor progression and immune evasion [229]. CD73 is expressed on approximately 75% of B cells, 50% of CD8+ T cells, 10% of CD4+ T cells, and 2–5% of NK cells [230]. B cells, Tregs, Th17 cells, NK cells, myeloid-derived suppressor cells (MDSC), endothelial cells, fibroblasts, and stromal and tumor cells can co-express CD39 and CD73. The overexpression of CD39 and CD73 is associated with worse prognoses in various cancers, including melanoma, lung cancer, colorectal cancer, and breast cancer [231]. Recent insights into their immunoregulatory roles have positioned them as promising targets for next-generation cancer immunotherapies.

**Figure 6:**
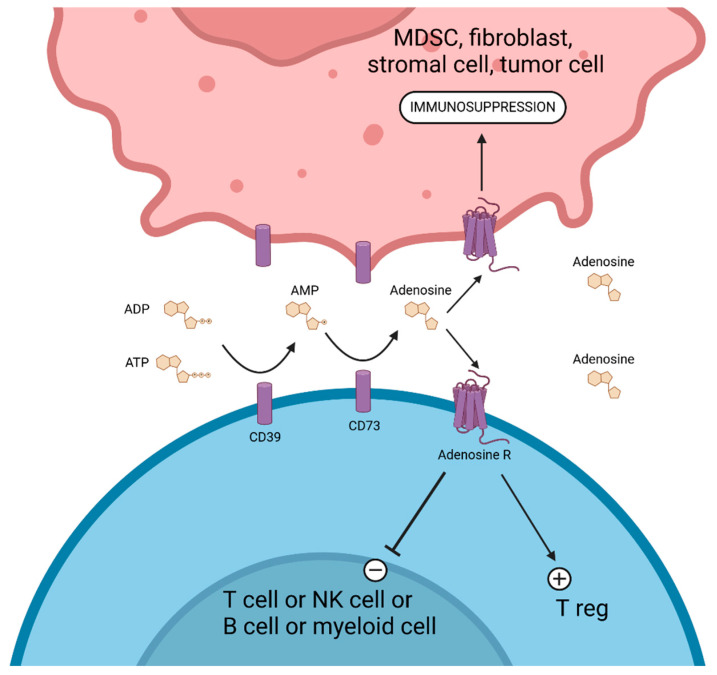
CD39/CD73/adenosine pathway. Adenosine R: adenosine receptor; AMP: adenosine monophosphate; ADP: adenosine diphosphate; ATP: adenosine triphosphate; MDSC: myeloid-derived suppressor cell.

Multiple mechanisms could explain the deleterious effect of the CD39/CD73 pathway on the antitumor immune response (Figure 6). Extracellular ATP (eATP), released by stressed or dying cells in the TME, could promote inflammation and immune activation via purinergic receptors like P2X7. CD39 and CD73 convert eATP into adenosine, dampening immune responses by binding adenosine receptors (A2A and A2B). This inhibits T cell proliferation, cytokine production, and effector functions while enhancing Treg activity. Adenosine signaling directly suppresses cytotoxic CD8+ T cells and NK cells, while promoting immune checkpoints like PD-1 and CTLA-4. CD39 and CD73 also enhance angiogenesis and metastasis by increasing VEGF and IL-8 production in the TME. CD39/CD73 activity skews macrophages from a pro-inflammatory M1 phenotype to an immunosuppressive M2 phenotype and could also impair DC maturation, reduce antigen presentation, and suppress pro-inflammatory cytokine production [232]. Published results from clinical trials with drugs targeting the CD39/CD73/adenosine pathway are summarized in Table 6.

### 8.1. CD39 Targeting

CD39’s enzymatic activity reduces eATP and converts ATP into ADP. High CD39 expression is observed in terminally exhausted T cells, notably tumor-specific exhausted T cells. Preclinical studies have shown that blocking CD39 mAbs or using inhibitors could impede tumor growth and improve CD8 antitumor immune response in various murine models of melanoma, lung carcinoma, and colon tumors. Combining CD39-blocking mAbs with aPD-1 showed synergistic effects, further enhancing tumor control and immune response, thus suggesting a rationale for testing this combination in patients.

#### IPH5201

IPH5201 is an aCD39 mAb that has been assessed in a phase I basket trial enrolling patients with advanced solid tumors in combination with durvalumab [233]. The combination reached a median PFS and OS of 1.4 and 8.2 months, respectively, but data have not been published yet, particularly regarding the mNSCLC population. The phase II MATISSE [234] trial is currently assessing this combination in a multicenter, single-arm study, combining neoadjuvant IPH5201, durvalumab, and platinum-doublet chemotherapy followed by surgery then 1-year of adjuvant IH5201 and durvalumab, in patients with resectable, early-stage (II to IIIA) NSCLC.

Other CD39-targeted mAbs have been evaluated in early-phase basket trials in advanced solid tumors: SRF617 [226] (NCT05177770), TTX-030 [235] (NCT06119217 for pancreatic cancer, NCT03884556, NCT04306900), and ES002023 (NCT05075564).

### 8.2. CD73 Targeting

CD73’s enzymatic activity transforms AMP into adenosine, a powerful immunosuppressive enzyme. aCD73 mAbs were shown to enhance pro-mitogenic effects of anti-CD3 mAbs on human T cells in vitro [236]. In animals, CD73-deficient mice also have increased antitumor immunity, and preclinical studies have shown synergy between aCD73 therapy and immunotherapy [237,238]. Targeting CD73 has also been shown to suppress tumorigenesis by affecting anti-apoptotic proteins [239] or suppressing tumor angiogenesis [240].

#### Oleclumab

Oleclumab is a first-in-class therapeutic aCD73 mAb, a human IgG1λ, tested in phase I alone or with durvalumab with no safety concerns [241]. Regarding the neoadjuvant setting, in the phase II NeoCOAST platform trial [219], 24 patients received a single cycle of oleclumab with durvalumab before surgery for stage IA3-IIIA NSCLC. With oleclumab on top of aPD-L1, the MPR rate increased from 11.1% to 19.0% and the pCR rate from 3.7 to 9.5%. In a more advanced setting, the phase II COAST study [220,221] (unresectable stage III NSCLC) assessed the addition of oleclumab to adjuvant durvalumab after chemoradiation. The addition of oleclumab to durvalumab increased the ORR from 23.9% with durvalumab monotherapy to 35.0% with the combination and prolonged mPFS from 7.3 months with durvalumab monotherapy to 21.1 months. The combination might also prolong OS (data not mature). The phase Ib/IIA PIONeeR study [224] tested durvalumab and oleclumab therapy but with mNSCLC patients who had already experienced disease progression after sequential or concomitant PD-(L)1 and platinum-based chemotherapy. The 12-week DCR reached with the combination was 0% vs. 54.5% with docetaxel, suggesting lower efficacy in this context, as with the monalizumab and durvalumab combination.

### 8.3. Adenosine Receptor Inhibitors

An alternative strategy to target adenosine pathways is to target adenosine receptors. Extracellular adenosine can activate four distinct P1 purinergic receptors, namely A1, A2a, A2b, and A3 adenosine receptors [242]. A1, A2a, and A3 are considered as high-affinity receptors for adenosine, while the A2b receptor has lower affinity. The A2a and A2b receptor antagonists demonstrated safety and clinical activity in solid tumors when combined with chemotherapy/immunotherapy.

#### 8.3.1. Etrumadenant

Etrumadenant is an orally bioavailable, selective, A2a and A2b receptor antagonist that has demonstrated safety and clinical activity in solid tumors when combined with chemo/immunotherapy. The ARC-7 [108] phase II trial (NCT04262856) contains an arm with the combination of zimberelimab, domvanalimab, and etrumadenant in treatment-naive patients with PDL1 > 50% (TPS) and no targetable oncogenic addiction. With the addition of etrumadenant and domvanalimab to the aPD-1, the ORR increased from 12% to 18%, and the median PFS increased from 5.4 to 10.9 months.

#### 8.3.2. Taminadenant

Taminadenant is an orally bioavailable, A2a receptor antagonist. In a phase I/Ib study [243], mNSCLC patients treated with at least one prior therapy received taminadenant with or without spartalizumab (aPD-1). The ORRs with the combination of taminadenant plus aPD-1 and with taminadenant monotherapy were 8.3% and 9.5%, respectively. In the taminadenant monotherapy arm, the median PFS and OS were 3.9 and 9.7 months, respectively, compared to 2.8 and 5.4 months, in the combination arm. These data suggest that targeting the adenosine pathway at various sites of the CD39/CD73/adenosine receptor axis might be a promising approach to enhance the antitumor immune response in the setting of immunotherapy combinations. Translational studies are needed to determine key biomarkers for selecting mNSCLC patients who would be more likely to respond to such therapies.

**Table 6:** Published data from clinical trials with drugs targeting the CD39/CD73/adenosine pathway.

aCD73 mAb	Type	Study	Phase	Setting	N	Intervention	1st Endpoint	Results with aCD73	Ref
**Oleclumab**	Human IgG1λ	NeoCOAST	II	Resectable, stage IA3-IIIA, neoadjuvant	83	Mona (aNKG2A) + durva (aPD-L1) vs. ole + durva vs. danvatirsen + durva vs. durva	MPR	MPR 19.0% with ole + durva vs. 11.1% with durva pCR 9.5% with ole + durva vs. 3.7% with durva Grade ≥ 3 TRAE 4.8% with ole + durva and 0% with durva	[219]
		COAST	II	Unresectable, stage III, after chemoradiation	189	Mona + durva vs. ole + durva vs. durva	ORR	ORR 35.0% with ole + durva, vs. 23.9% with durva mPFS 21.1 mo with ole + durva vs. 7.3 mo with durva (HR 0.59, 95% CI 0.37, 0.93) mOS NR with ole + durva vs. 40.9 mo with durva (HR 0.69, 95% CI 0.40, 1.20) Grade ≥ 3 TEAE 33.9% with ole + durva vs. 34.8% with durva	[220,221]
		PIONeeR	Ib/IIA	Metastatic, 2nd or 3rd line, after aPD-(L)1	114	Mona + durva vs. ole + durva vs. ceralasertib + durva vs. savolitinib + durva vs. docetaxel	12 w DCR	12 w DCR 0% with ole + durva (prematurely closed due to lack of efficacy) vs. was 54.5% with docetaxel No safety data	[224]
**Adenosine R I**	**Type**	**Study**	**Phase**	**Setting**	**N**	**Intervention**	**1st Endpoint**	**Results with Adenosine R I**	**Ref**
**Etrumadenant**	Selective dual antagonist of A_2a_ and A_2b_	ARC-7	II	Metastatic, 1st line, PD-L1 ≥50%	151	Domva (aTIGIT) + zimbe (aPD-1) + etrumadenant vs. domva + zimbe vs. zimbe	ORR, PFS	ORR 18% with domva + zimbe + etrumadenant vs. 12% with zimbe mPFS 10.9 mo with domva + zimbe + etrumadenant vs. 5.4 mo with zimbe (HR 0.65, 95% CI 0.37–1.1) Grade ≥ 3 TEAE 52% with domva + zimbe + etrumadenant vs. 58% with zimbe	[108]
**Taminadenant**	A_2a_ antagonist	Chiappori et al.	I/Ib	Metastatic, ≥2nd line	50	Taminadenant vs. taminadenant + sparta (aPD-1)	Safety	ORR 9.5% with taminadenant vs. 8.3% with taminadenant + sparta mPFS 3.9 mo with taminadenant vs. 2.8 mo with taminadenant + sparta mOS 9.7 mo with taminadenant vs. 5.4 mo with taminadenant + sparta Grade ≥ 3 TRAE 16.0% with taminadenant vs. 36.0% with taminadenant + sparta	[243]

mAb: monoclonal antibody; N: number of patients included; mona: monalizumab; durva: durvalumab; ole: oleclumab; MPR: major pathological response; pCR: pathological complete response; TRAE: treatment-related adverse events; ORR: objective response rate; mPFS: median progression-free survival; mo: months; HR: hazard ratio; CI: confidence index; mOS: median overall survival; TEAE: treatment-emergent adverse events; 12 w DCR: 12-week disease control rate; adenosine R I: adenosine receptor inhibitor; domva: domvanalimab; zimbe: zimberelimab; PFS: progression-free survival; sparta: spartalizumab.

## 9. Conclusions

Immunotherapy has transformed the landscape of NSCLC treatment, yet the limitations of PD-1/PD-L1 targeting underscore the need for novel strategies. Emerging immune checkpoints, such as CTLA-4, TIGIT, LAG-3, and TIM-3, provide promising avenues to overcome resistance and enhance antitumor immunity. Advances in bispecific and Fc-engineered antibodies offer additional potential for therapeutic synergy and precision. Going forward, a deeper understanding of the tumor microenvironment, alongside refined biomarkers for patient selection, will be crucial in optimizing these approaches and expanding their impact on clinical outcomes.
